# PPARα-Dependent Modulation by Metformin of the Expression of OCT-2 and MATE-1 in the Kidney of Mice

**DOI:** 10.3390/molecules25020392

**Published:** 2020-01-17

**Authors:** Adriano Cleis Arruda, Mauro Sérgio Perilhão, Warley Almeida Santos, Marcos Fernandes Gregnani, Alexandre Budu, José Cesar Rosa Neto, Gabriel Rufino Estrela, Ronaldo Carvalho Araujo

**Affiliations:** 1 Department of Biophysics, Federal University of São Paulo, 04039032 São Paulo, Brazil; adriano.arruda@unifesp.br (A.C.A.); m.perilhao@unifesp.br (M.S.P.); wharleysan@gmail.com (W.A.S.); mgregnani@hotmail.com (M.F.G.); alexandre.budu@unifesp.br (A.B.); 2 Department Cell Biology and Development, Institute of Biomedical Sciences, University of São Paulo, 05508000 São Paulo, Brazil; josecesar23@hotmail.com; 3 Department of Clinical and Experimental Oncology, Discipline of Hematology and Haematotherapy, Federal University of São Paulo, 04037002 São Paulo, Brazil; g.estrela@unifesp.br

**Keywords:** metformin, PPARα, MATE-1, OCT-2, transcription, kidney

## Abstract

Metformin is the first-line drug for type 2 diabetes mellitus control. It is established that this drug traffics through OCT-2 and MATE-1 transporters in kidney tubular cells and is excreted in its unaltered form in the urine. Hereby, we provide evidence that points towards the metformin-dependent upregulation of OCT-2 and MATE-1 in the kidney via the transcription factor proliferator-activated receptor alpha (PPARα). Treatment of wild type mice with metformin led to the upregulation of the expression of OCT-2 and MATE-1 by 34% and 157%, respectively. An analysis in a kidney tubular cell line revealed that metformin upregulated PPARα and OCT-2 expression by 37% and 299% respectively. MK-886, a PPARα antagonist, abrogated the OCT-2 upregulation by metformin and reduced MATE-1 expression. Conversely, gemfibrozil, an agonist of PPARα, elicited the increase of PPARα, OCT-2, and MATE-1 expression by 115%, 144%, and 376%, respectively. PPARα knockout mice failed to upregulate both the expression of OCT-2 and MATE-1 in the kidney upon metformin treatment, supporting the PPARα-dependent metformin upregulation of the transporters in this organ. Taken together, our data sheds light on the metformin-induced mechanism of transporter modulation in the kidney, via PPARα, and this effect may have implications for drug safety and efficacy.

## 1. Introduction

Recent progress has been made in understanding the role of membrane transporters in drug safety and efficacy. Presently, there are more than 400 membrane transporters, divided in two major families the ATP-binding cassette and the solute carrier (SLC) family. These transporters are key players in drug accumulation within cells, therapeutic efficacy, drug toxicity, and drug-drug interactions therefore exerting an important role in pharmacokinetics and pharmacodynamics [1,2]. The organic cation transporters belong to the SLC family; among them, OCT-1, OCT-2, OCT-3, and MATE-1 are abundant in the kidney, where they control de efflux and influx of endogenous organic cations, drugs, and toxins [2]. A plethora of drugs are carried through transporters of the OCT and MATE family, including metformin, the first-line drug for type 2 diabetes *mellitus* control [3]. Renal excretion consists of three steps: glomerular filtration, tubular secretion and reabsorption [3,4].

Upon reaching the kidney, due to its hydrophilic nature, metformin is not able to enter the cytosol via diffusion through the plasma membrane; it enters the proximal tubule kidney cells from the blood via specific cation transporters, namely OCT-2 [5,6] located in the proximal tubule basolateral membrane. Metformin is then secreted to the urine by the multidrug and toxin extrusion protein MATE-1, located in the luminal membrane of the proximal tubule [7,8]. OCT-2 is of particular importance in drug transport, since it was shown to be a target for protective interventions against drug nephrotoxicity [9].

Studies have suggested that the peroxisome proliferator-activated receptor alpha (PPARα) regulates the transcription of several transporters, as the ATP-binding cassette-transporter A1, the fatty acid transport protein and the nucleoside transporter hENT1 [10,11,12]. Similarly, Nie et al., 2005 demonstrated that PPARα and -γ agonists significantly increased gene expression of hepatic OCT-1, in mice and H35 cells. [13]. In another study, Oda et al., 2014 suggest that PPARα controls OCT-2 expression via a circadian cycle-dependent mechanism, as there is a concomitant increase of OCT-2 and PPARα during the light period [10]. The PPARα-dependent modulation of MATE-1, on the other hand, was not explored in depth. Moreover, the modulation of OCT-2 and MATE-1 transporter expression by metformin in the kidney is poorly understood and may have implications for the safety and efficacy of the use of this drug. Therefore, we sought to investigate the effect of metformin upon OCT-2 and MATE-1 transcription in this organ.

## 2. Results

### 2.1. Metformin Administration is Capable of Modulating OCT-2, MATE-1, and PPARα Expression at the mRNA Level in the Kidney

**Figure 1:**
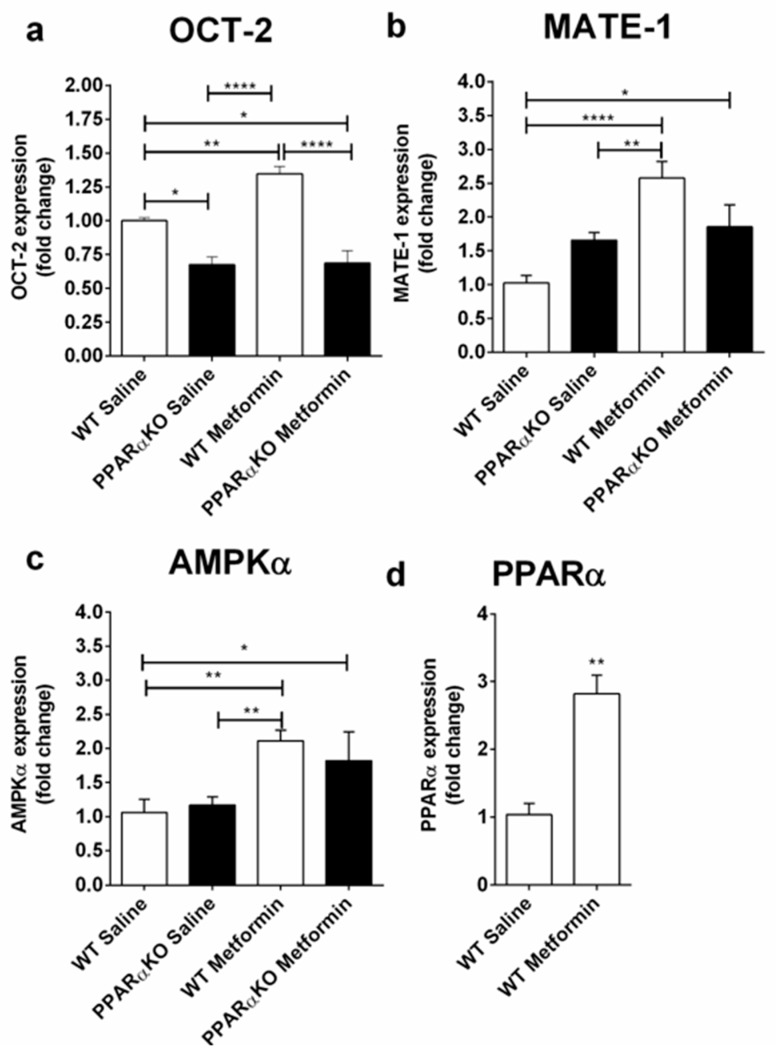
Real-Time PCR of OCT-2 (**a**), MATE-1 (**b**), AMPKα (**c**) and proliferator-activated receptor alpha (PPARα) (**d**) in the kidney of mice treated with saline or metformin (300 mg/kg) for 30 days. Data was compared by Two-Way ANOVA and Tukey multiple comparisons test (**a**–**c**), or unpaired, nonparametric Mann-Whitney test (**d**); * *p* < 0.05, ** *p* < 0.01, *** *p* < 0.001, **** *p* < 0.0001. Data are presented as Mean ± S.E.M.

Animals were euthanized after 30 days and the kidney and blood were collected. No difference in urea concentration in the plasma was observed between the saline-treated wild type mice (WT saline) and the metformin-treated wild type animals (WT metformin) or between the PPARα knockout groups (PPARαKO, Figure S1a). No difference in blood lactate levels was found between the WT saline and WT metformin groups (Figure S1b). Upon the assessment of OCT-2 and MATE-1 expression in the kidney, a rise in the WT metformin relative to the WT saline group was observed (Figure 1a,b). Conversely, metformin administration was not able to elicit a statistically significant increase in OCT-2 and MATE-1 expression in PPARαKO mice, and OCT-2 expression levels in PPARαKO mice treated with saline (PPARKO saline) were lower relative to WT saline mice (Figure 1a,b). The treatment with metformin was able to modulate the AMPKα in the kidney, in both WT and PPARαKO relative to WT saline group (Figure 1c). A non-statistically significant difference of 65% was observed between PPARαKO metformin and PPARαKO saline group (Figure 1c). Furthermore, the metformin treatment was able to increase the expression of PPARα at the mRNA level in WT mice (Figure 1d). These results prompted us to speculate that metformin modulates the expression levels of OCT-2 and MATE-1 in tubular kidney cells via the transcription factor PPARα.

### 2.2. Metformin Treatment Modulates PPARα, OCT-2, and MATE-1 Expression at the mRNA Level in the MM55.K Cell Line

**Figure 2:**
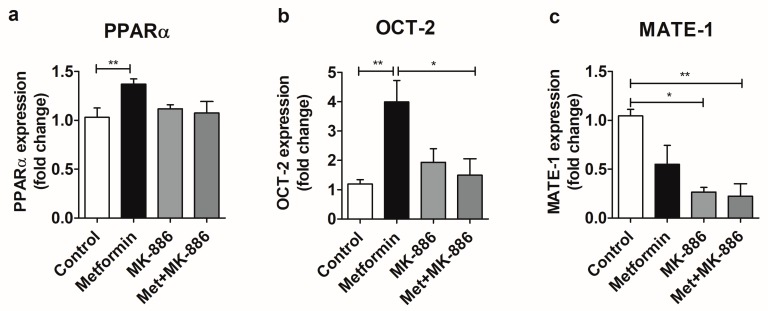
Modulation of PPAR alpha (**a**), OCT-2 (**b**), and MATE-1 (**c**) expression by metformin (50 µM). MK-886 was used at 25 µM. Cells were incubated for 24 h with the compounds. Data was compared by the nonparametric Kruskal-Wallis test, * *p* < 0.05, ** *p* < 0.01. Data is presented as Mean ± S.E.M. Met, metformin.

Aiming to analyze whether metformin modulates the expression of OCT-2 and MATE-1 in kidney tubular cells via PPARα, we conducted experiments with the mouse cell line MM55.K. Treatment with metformin (50 µM) evoked the upregulation of PPARα and OCT-2 in tubular cells (Figure 2a,b); however treatment with metformin failed to increase the expression of MATE-1 (Figure 2c). Interestingly, MK-886 (25 µM) abrogated the metformin-induced upregulation of OCT-2 and PPARα (Figure 2a,b) and downregulated the expression of MATE-1 (Figure 2c).

### 2.3. Gemfibrozil Treatment Modulates PPARα and OCT-2 Expression at the mRNA Level in the MM55.K Cell Line

**Figure 3:**
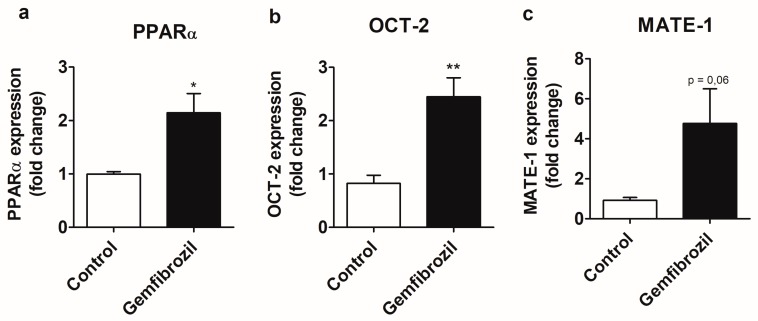
Modulation of PPARα (**a**), OCT-2 (**b**), and MATE-1 (**c**) expression by gemfibrozil (100 µM). Cells were incubated for 24 h with the compounds. Data was compared by the unpaired, nonparametric Mann-Whitney test, * *p* < 0.05, ** *p* < 0.01. Data is presented as Mean ± S.E.M.

In order to further establish the involvement of PPARα in the modulation of MATE-1 and OCT-2 in kidney tubular cells we conducted experiments with the mouse cell line MM55.K using a known PPARα agonist, gemfibrozil. The PPARα agonist was able to increase the expression of PPARα by 115% and OCT-2 by 144% (Figure 3a,b) in a statistically significant manner while there was a 376% increase in MATE-1 expression relative to the control (Figure 3c).

## 3. Discussion

In the kidney, OCT-2 and MATE-1 are the major transporters for secretion of cationic drugs in the urine. Metformin has a great affinity for OCT-2 and MATE-1 [3]. Several studies have addressed the importance of the expression levels of metformin transporters as they may modify pharmacokinetics and pharmacodynamics of this drug, thus altering its efficacy or evoking toxic effects in the organism [6,14,15,16]. Therefore, assessing the effect of the administration of metformin on its transporter expression may help to explain the drug efficacy and safety. Studying the mechanisms of the modulation of metformin transporter expression in the kidney is particularly important, as this organ is responsible for the excretion of metformin, a drug that is not metabolized elsewhere [17].

The kidney is the main organ involved with drug elimination; 25% of the human use-approved medicines are eliminated in an unaltered form in the urine while nephrons, the functional units of the kidney, execute glomerular filtration, tubular secretion, and reabsorption [4]. Glomerular filtration is passive, while secretion and reabsorption are based on transporters located at the basolateral and luminal membranes [4,18]. These transporters are predominantly expressed in the proximal tubule and are responsible for carrying the drugs from the blood to the urine [4,18]. The main transporters involved with tubular secretion are OCT-2, located at the basolateral membrane, and MATE-1 and MATE-2, located at the luminal membrane [3,4,7,18,19,20]. Alterations in OCT-2 and MATE-1 activity may impact the elimination of drugs, leading to its accumulation within the cells of the proximal tubule and thus evoking nephrotoxicity and lactic acidosis [16,21].

Previous work established that the OCT-1 and OCT-2 transporters can be modulated by the peroxisome proliferator-activated receptor alpha (PPARα) [10,13,22]. Our work has added more layers of detail to the latter mechanism, establishing that metformin was able to increase the expression of OCT-2 and MATE-1 via PPARα in a specific organ, namely the kidney of mice (Figure 1). This conclusion is supported by the observations that PPARαKO mice did not display an increase in OCT-2 and MATE-1 expression upon metformin treatment as did WT mice (Figure 1a,b), a statistically significant decrease in OCT-2 expression was observed in PPARαKO saline mice relative to the WT saline counterparts (Figure 1a). When we brought the analysis to the cellular level, employing a kidney tubular cell line (MM55.K) we observed that metformin led to the upregulation of PPARα and OCT-2. Interestingly, incubation MK-886 led to the abrogation of the OCT-2 and PPARα metformin-induced expression increase, indicating that metformin acts via the transcription factor PPARα to induce the expression of these two genes (Figure 2a,b). MATE-1 expression was not altered by metformin treatment in the tubular cells analysis, however MK-886 was able to decrease its expression relative to the control (Figure 2c), suggesting that while some player in metformin-induced MATE-1 expression might be missing in these cells, PPARα is still able to control its expression.

Metformin activates AMPK via two separate mechanisms, directly, by activating the kinase via phosphorylation at Thr-172 (α-subunit), and indirectly by inhibiting the mitochondrial respiratory chain complex 1, causing an increase in the cellular AMP:ATP ratio, which leads to AMPKα phosphorylation [17,23]. AMPK activity can lead to the suppression of oxidative stress, apoptosis, and accumulation of damaged proteins and organelles [24]. Our study shows that the treatment with metformin increases the expression AMPKα in the kidney of WT and PPARαKO mice, hinting towards protective effects of metformin in this organ (Figure 2c).

Further supporting the role of PPARα in its own expression and in the expression of OCT-2 and MATE-1, gemfibrozil, a PPARα agonist, was able to increase the expression of these genes (Figure 3a–c). In agreement with these results, an in silico study that integrates experimental data from high throughput gene expression screening predicts PPARα response elements (PPRE) in the PPARα and MATE-1 genes [25]. There is no prediction of PPREs in OCT-2 by Fang et al. [25], however it is worth mentioning that only 5 kb regions upstream and downstream of the genes were analyzed and there are PPREs predicted in the intron of genes [26]. Indeed, PPREs were found in the first intron of the murine OCT-2 and a reporter gene assay and gel shift assay demonstrated PPARα-dependent transcription induction and binding to this region [27].

**Figure 4:**
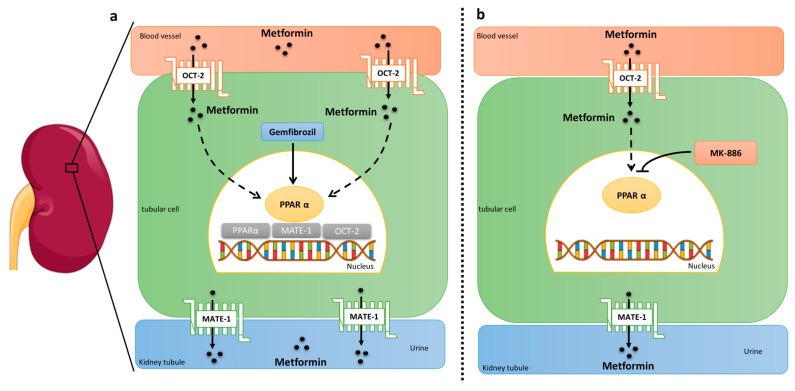
Summary of the effects of metformin in the modulation of OCT-2 and MATE-1 transporters via PPARα. (**a**) Metformin was able to increase the expression of PPARα, OCT-2, and MATE-1. Gemfibrozil, a PPARα agonist, increases the expression of PPARα, OCT-2, and MATE-1. (**b**) The PPAR α antagonist, MK-886, abrogated the metformin-induced regulation of PPARα and OCT-2 and reduced the expression of MATE-1.

Our results support the metformin induces a PPARα-dependent upregulation of OCT-2, MATE-1 and PPARα expression in tubular kidney cells (Figure 4). Taken together, our data suggests that the modulation of drug transporters in the kidney by metformin contributes for the safety of the drug administration at 300 mg/kg in mice.

## 4. Materials and Methods

### 4.1. Animals

Wild type (WT) and PPARα knockout (PPARαKO mice, B6; 129S4-*Ppara^tm1Gonz^*/J, Jackson laboratory) male mice (10–12 weeks old) of the C57BL/6 strain were employed in our study. The animals were maintained under controlled temperature conditions (22 °C) and with a light/dark period of 12 h/12 h, with water and food ad libitum. All the procedures were approved by the Federal University of São Paulo Animal Ethics Committee (*CEUA*) under the number 8549250416, approval date 17 April 2016.

### 4.2. Experimental Design in Mice

Metformin, diluted in 0.9% (*m*/*v*) NaCl, was administered daily at 300 mg/kg via gavage for 30 days while 0.9% NaCl (vehicle) was administered to the control group. Animals were divided into 4 groups: Saline (WT, *n* = 6), Saline (PPARαKO, *n* = 5), Metformin (WT, *n* = 7), Metformin (PPARαKO, *n* = 6). Lactate was measured weekly in WT animals with the Accutrend Plus instrument (Roche, Basel, Swizerland). Animals were euthanized after 30 days of the first metformin administration and the kidneys were collected and kept frozen at −80°C until the extraction of RNA and cDNA synthesis. Blood was also collected and plasma was used to measure urea using a kit and according to the manufacturer’s instructions (Urea CE, Labtest, Brazil, #27-500).

### 4.3. Experimental Design in MM55.K Cells

The mouse kidney tubular cell line MM55.K (ATCC ref. CRL-6436) was maintained in DMEM (Gibco, #11965092) supplemented with 10% fetal bovine serum (FBS), at 37 °C, 5% CO_2_ atmosphere and split with trypsin/EDTA solution. Cells were plated onto 6-well plates (3 × 10^5^ cells/well) and, after 24 h of culture, treated with gemfibrozil (100 µM), MK-886 (25 µM, Cayman Chemical, #10133), a PPARα antagonist, and/or metformin (50 µM) in DMEM containing 10% FBS. After 24 h in culture, cells were briefly washed with PBS (137 mM NaCl, 2.7 mM KCl, 8.1 mM Na_2_HPO_4_, 1.5 mM KH_2_PO_4_). RNA extraction, cDNA synthesis, and Real-Time PCR were then conducted.

### 4.4. RNA Extraction and Real-Time PCR

RNA was extracted with Trizol (#15596026, Thermo) according to the manufacturer’s instructions after processing approximately 1/3 of a kidney in a Precellys (Bertin Instruments, Montigny-le-Bretonneux, France). RNA integrity was assessed in agarose gels. Synthesis of cDNA was conducted with High Capacity cDNA Reverse Transcription kit (Thermo, #4368814) using random primers and according to the manufacturer’s instructions. The Real-Time PCR reaction was performed using 50ng of cDNA and Hot FirePol Evagreen qPCR Mix, ROX (Solis Biodyne, #08-24-00001), 10 µL reaction volume in QuantStudio3 equipment (Applied Biosystems). The following primers were employed (5′-3′): MATE-1, forward: AGGCCAAGAAGTCCTCAGCTATT; MATE-1, reverse: ACGCAGAAGGTCACAGCAAA; OCT-2, forward: AGCCTGCCTAGCTTCGGTTT; OCT-2, reverse: TGCCCATTCTACCCAAGCA; PPARα, forward: ATGCCAGTACTGCCGTTTTC; PPARα, reverse: TTGCCCAGAGATTTGAGGTC; AMPKα, forward: GCCTGGAACATACCTAACAC, reverse: TTGCCAGACTGAACCAAACAC, 18S, forward: CCTGCGGCTTAATTTGACTC; 18S, reverse: AAGACAAATCGCCTCCACCAAC. Relative quantification was performed using the 2^−ΔΔCt^ method using 18S as the reference gene.

### 4.5. Statistical Analysis

GraphPad Prism v8.2.1 was used for Statistical Analysis. Data is shown as Mean ± S.E.M. Data was compared by Two-Way ANOVA and Tukey multiple comparisons test, unpaired, nonparametric Mann-Whitney test or Kruskal-Wallis test. Statistical significance was considered for *p* < 0.05.

## Supplementary Materials

The following are available online, Figure S1: Measurement of plasma urea (**a**) in wild type (WT) and PPARα knockout (PPARαKO) mice and blood lactate (**b**) in wild type (WT) mice treated with 300 mg/kg of metformin once a day for 30 days.
