# Supplementary material for: PPARα-Dependent Modulation by Metformin of the Expression of OCT-2 and MATE-1 in the Kidney of Mice
Source: Molecules. 2020 Jan 17;25(2):392. doi: 10.3390/molecules25020392 (PMC7024194; doi:10.3390/molecules25020392)
Supplement: Supplementary file 1 [file molecules-25-00392-s001.pdf]

Supplemental Materials

Article

PPARα-Dependent Modulation by Metformin of the Expression of OCT-2 and MATE-1 in the Kidney of Mice

Adriano Cleis Arruda [1], Mauro Sérgio Perilhão [1], Warley Almeida Santos [1], Marcos Fernandes Gregnani [1], Alexandre Budu [1], José Cesar Rosa Neto [2], Gabriel Rufino Estrela [3] and Ronaldo Carvalho Araujo [4,*]

[1] Department of Biophysics, Federal University of São Paulo, 04039032 São Paulo, Brazil; adriano.arruda@unifesp.br (A.C.A.); m.perilhao@unifesp.br (M.S.P.); wharleysan@gmail.com (W.A.S.); mgregnani@hotmail.com (M.F.G.); alexandre.budu@unifesp.br (A.B)

[2] Department Cell Biology and Development, Institute of Biomedical Sciences, University of São Paulo, 05508000, São Paulo, Brazil; josecesar23@hotmail.com

[3] Department of Clinical and Experimental Oncology, Discipline of Hematology and Haematotherapy, Federal University of São Paulo, 04037002 São Paulo, Brazil; g.estrela@unifesp.br

[4] Department of Biophysics, Federal University of São Paulo, 04039032 São Paulo, Brazil; araujo.ronaldo@unifesp.br

*Correspondence: araujo.ronaldo@unifesp.br; Tel.: (+55-11-5576-4859)

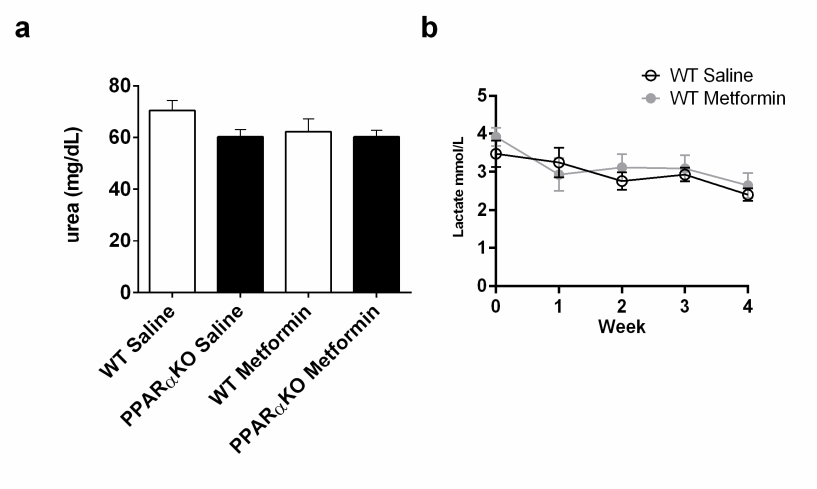

Figure S1. Measurement of plasma urea (**a**) in wild type (WT) and PPARα knockout (PPARαKO) mice and blood lactate (**b**) in wild type (WT) mice treated with 300 mg/kg of metformin once a day for 30 days. Data was compared by Two-Way ANOVA and Tukey multiple comparisons test (**a**), or unpaired, nonparametric Mann-Whitney test (**b**). Data is presented as Mean ± S.E.M.
